# CCL7 playing a dominant role in recruiting early OCPs to facilitate osteolysis at metastatic site of colorectal cancer

**DOI:** 10.1186/s12964-022-00867-7

**Published:** 2022-06-17

**Authors:** He Yang, Li Jian, Qian Jin, Kang Xia, Wang Cai-ru, Sheng Jun, Huang Chen, Wang Wei, Song Ben-jing, Li Shi-hong, Long Shi-wei, Wu Juan, Zheng Wei

**Affiliations:** 1 Department of Orthopedics, General Hospital of Western Theater Command, Rongdu Avenue No. 270, Chengdu, 610000 People’s Republic of China; 2 Chengdu Medical College, Rongdu Avenue No. 601, Chengdu, 610000 People’s Republic of China; 3 Department of Pharmacy, General Hospital of Western Theater Command, Rongdu Avenue No. 270, Chengdu, 610000 People’s Republic of China

**Keywords:** Bone metastasis, CRC, Osteoclast precursors, CCL7, CCR1

## Abstract

**Background:**

Chemoattractant is critical to recruitment of osteoclast precursors and stimulates tumor bone metastasis. However, the role of chemoattractant in bone metastasis of colorectal cancer (CRC) is still unclear.

**Methods:**

Histochemistry analysis and TRAP staining were utilized to detect the bone resorption and activation of osteoclasts (OCs) after administration of CCL7 neutralizing antibody or CCR1 siRNA. qRT-PCR analysis and ELISA assay were performed to detect the mRNA level and protein level of chemoattractant. BrdU assay and Tunel assay were used to detect the proliferation and apoptosis of osteoclast precursors (OCPs). The migration of OCPs was detected by Transwell assay. Western blots assay was performed to examine the protein levels of pathways regulating the expression of CCL7 or CCR1.

**Results:**

OCPs-derived CCL7 was significantly upregulated in bone marrow after bone metastasis of CRC. Blockage of CCL7 efficiently prevented bone resorption. Administration of CCL7 promoted the migration of OCPs. Lactate promoted the expression of CCL7 through JNK pathway. In addition, CCR1 was the most important receptor of CCL7.

**Conclusion:**

Our study indicates the essential role of CCL7-CCR1 signaling for recruitment of OCPs in early bone metastasis of CRC. Targeting CCL7 or CCR1 could restore the bone volume, which could be a potential therapeutical target.

**Video Abstract**

**Supplementary Information:**

The online version contains supplementary material available at 10.1186/s12964-022-00867-7.

## Background

Colorectal cancer (CRC) is one of the three major cancers that lead to cancer-related death in the world. With the improvement of living standards and the change of lifestyle, the incidence of CRC is on the rise. Notably, bone metastasis of CRC present with a relative lower incidence compared to other metastatic sites, however, the prognosis is worse [1, 2].

The various cells that make up bone tissue are in a dynamic balance, in which osteoblasts and osteoclasts mainly affect bone remodeling. CRC bone metastasis can lead to a series of adverse consequences, including pathological fracture, hypercalcemia, nerve compression syndrome, etc. [3, 4]. In the occurrence of pathological fracture and hypercalcemia, this dynamic balance is broken, and osteoclasts are abnormally activated, resulting in excessive bone absorption [5]. Previous studies have shown that osteoclast precursors (OCPs) are derived from monocytes/macrophages [6]. In the tumor microenvironment, the interaction between osteoclasts and tumor cells is an important link in the "vicious cycle" of tumor bone metastasis. Current studies have shown that in breast cancer, prostate cancer and other tumors, some chemokines can cause bone metastasis by affecting the recruitment of macrophages and osteoclasts [7, 8]. However, in the microenvironment of CRC bone metastasis, the mechanism and target of osteoclast regulation remain largely unclear.

A large number of studies show that chemokines and chemokine receptors can influence tumor growth, invasion and metastasis, which CCL7 is an important part of the monocyte chemotactic factor, in the normal body can be expressed in a variety of cells, the pathological conditions can be expressed in tumor cells [9–11]. Especially, it is highly expressed in renal cancer, gastric cancer, colorectal cancer and squamous cell [11–13]. However, there are few studies on the mechanism of CCL7 in colon cancer bone metastasis, and few studies on the effect of osteoclastic precursor cells in the microenvironment of colon cancer bone metastasis.

In this study, we explored that CCL7 played a dominant role in recruitment of OCPs in bone metastasis of CRC. In addition, lactate, which was abundant in CRC microenvironment, could upregulate the expression of CCL7 through activating JNK/c-Jun pathway, but not p38 MAPK pathway. Furthermore, MC-38 cells, a CRC cell line, could upregulate CCR1 in OCPs, one receptor of CCL7, which facilitated migration of early OCPs. Blockage of CCL7 or CCR1 could significantly attenuated the osteolysis in bone metastasis of CRC and restore the trabecular area. Our findings provide a new sight in regulation of OCPs in bone metastasis of CRC, which could be a potential therapeutical target for treating bone resorption.

## Methods

### Animal experiments

All animal experiments and procedures were approved by the Institutional Animal Care and Use Committee at General hospital of Western Theater Command. Because numerous studies reported that estrogen as well as its receptor largely impacted metastasis and progression of colorectal cancer cells [14–16], to eliminate this potential confounder, only male mice were used in this study. C57BL/6 male mice at 10 to 12 weeks were used for experiments. Mice were bred in house with free access to a normal chow diet and water, at a constant temperature (22 ± 2 °C) with a 12 h dark/light cycle. To establish the bone metastasis model of MC-38 cells, 500,000 of MC-38 cells were injected into tibia following standard procedures following previous method [17]. Briefly, making knee to be visible and accessible. Inserting the needle under patella into the anterior intercondylar area in top of tibia. Then penetrating of tibial growth plate and injecting 10 μl of cell solution. For blockage of endogenous CCL7, 20 μg of CCL7 neutralizing antibody (nAb) was injected intratibially twice every week until harvest. To test the treatment effect of CCR1 siRNA in vivo, 10 μg CCR1 siRNA (siCCR1) or negative control (scRNA) were mixed with INVI DNA RNA Transfection reagent (Invigentech, USA) for 15 min at room temperature (RT) according to the manufacturer’s instructions, and then was injected intratibially twice a week until harvest. The sequence for siCCR1: siRNA-1: sense(5′–3′): GGCAUCAUCACCAGUAUUATT and antisense(3′–5′): UAAUACUGGUGAUGAUGCCTT; siRNA-2: sense(5′–3′): GCCUCUGUUAGUCAUGAUATT and antisense(3′–5′): UAUCAUGACUAACAGAGGCTT.

### FACS and cell culture

CD115(+) early osteoclast precursors (thereafter named CD115(+) OCPs) or other cells in bone marrow were isolated as described in our previous studies. Briefly, bone marrow was rinsed out with sterile PBS. After filtered and centrifuged, cells from bone marrow were cultured in primary antibodies for 30 min on ice in dark. Then, the stained cells were sorted by FACSAria III (BD Biosciences). Early OCPs were labelled with anti-mouse CD115-APC (Biolegend), anti-mouse RANK-PE (Biolegend). Neutrophils, eosinophils or T cells were labelled with anti-mouse CD45 conjugated with Percp-Cy5.5 (Biolegend), anti-mouse CD11b conjugated with AF700 (Biolegend), anti-mouse Ly6G conjugated with APC-Cy7 (Biolegend), anti-mouse Siglec F conjugated with PE (BD Biosciences) or anti-mouse CD3 conjugated with PE (Biolegend). Bone marrow-derived mesenchymal stem cells (BMMSCs) were labelled with anti-mouse CD31 conjugated with APC (Biolegend), anti-mouse CD45 conjugated with APC (Biolegend), anti-mouse Ter119 conjugated with APC (Biolegend), anti-mouse Sca-1 conjugated with APC-Cy7 and anti-mouse Pdgfrα conjugated with PE (Biolegend).

Isolated CD115(+) OCPs were cultured in α-minimal essential medium (MEM) supplemented with 10% FBS and 1% Penicillin–streptomycin solution and 50 ng/mL colony stimulating factor (M-CSF, Abcam) for 48 h. Then, cells were stimulated with 50 ng/mL M-CSF and 100 ng/mL Receptor Activator of Nuclear Factor-κ B Ligand (RANKL) (Biolegend). The culture medium was changed every two days. In some experiments,

MC-38 cells were cultured in Dulbecco's modified Eagle's medium (DMEM) supplemented with 10% FBS and 1% Penicillin–streptomycin solution. For collecting conditional medium (CM), when MC-38 cells reached 80% confluence, the culture medium was replaced with DMEM for culturing additional 24 h. The culture medium was collected and centrifuged at 2000 rpm/min to remove cells and debris. Then, supernatant was collected and stored at -80 ℃.For treatment with MC-38 CM, MC-38 CM was added into culture medium at a ratio of 1:1 to treat for 48 h.

To treat with CCL7, recombinant CCL7 (100 ng/mL) was added into culture medium after early OCPs reached 80% confluence and treated for 48 h. To downregulate target genes, siRNA or scRNA was mixed with transfection reagent (Invigentech) for 15 min, and the mixture was added into culture medium incubated for 24 h. The sequences for siRNAs were listed as below: CCR1 siRNA, c-Fos siRNA, c-Jun siRNA. For treatment with lactate and inhibitors, 5 × 10^5^ CD115(+) early OCPs were cultured in 12-well plates. After reach 80% confluence, lactate was added at a concentration of 100 μg/mL in the presence of MC-38 CM or not, the cells were stimulated for 24 h. To inactive JNK pathway or p38 MAPK pathway, SP600125 (50 nM) or SB203582 (25 μM) was added to treat early OCPs for 8 h following by stimulated with lactate for 24 h. To inhibit ERK pathway, early OCPs were treated with 1 µM AZD0364 for 8 h in the presence of MC-38 CM.

### Cell migration assay

Cell migration assay was performed using Transwell-24 well permeable support plates with an 8.0-μm pore size membrane (Corning, USA). To detect the effect of CCL7 on OCPs migration, early OCPs from normal bone marrow were seeded on upper chamber at a density of 1 × 10^5^ cells. Recombinant CCL7 protein (100 ng/mL) was added into culture medium in lower chamber. To test whether CCL7 regulated the migration of OCPs through CCR1, early OCPs were transfected with CCR1 siRNAs, then the cells were seeded on upper chamber described as above.

To verify whether blockage of CCL7 could influence the OCPs migration, early OCPs from bone marrow in normal mice (Day 0) or at 10 days post injection of MC-38 cells (D10) treated with/without 10 μg/mL CCL7 nAb were seeded in lower chamber at a density of 3 × 10^5^. Early OCPs (1 × 10^5^ cells/well) isolated from normal bone marrow were seeded in the upper chamber. The chamber was incubated at 37 °C for 12 h. The cells were stained with crystal violet after culturing for 12 h. Then, samples were treated with 33% acetic acid for discoloration. Then, the absorbency at 570 nm was detected.

### RT-PCR analysis

Total RNA was isolated and performed using TRIzol reagent. RNA was reversely transcribed into cDNA by using RevertAid First Strand cDNA Synthesis kit (Thermo Fisher Scientific) following the manufacturer’s procedures. The mRNA levels were normalized to GAPDH. Relative target gene expression was calculated using the 2-ΔΔCq method. The primer sequences used for PCR were listed as below: GAPDH (RE: TGTAGACCATGTAGTTGAGGTCA; FW: AGGTCGGTGTGAACGGATTTG), CCL7 (RE: CTCGACCCACTTCTGATGGG; FW: CCCTGGGAAGCTGTTATCTTCAA), CCL2 (RE: GCTTGGTGACAAAAACTACAGC; FW: CACTCACCTGCTGCTACTCA), CCL6 (RE: CTGCCCTCCTTCTCAAGCAA; FW: TCAAGCCGGGCATCATCTTT), CCR1 (RE: TCCTTTGCTGAGGAACTGGTC; FW: ACTCTGGAAACACAGACTCAC), CCR2 (RE: ACTTGCATGGCATTTACAGGT; FW: ACTCCTGGCTGGAAAAGAAGA), CCR3 (RE: TGCCATTCTACTTGTCTCTGGT; FW: TGATGTTTACTACCTGACTGGTG).

### Western blotting

Proteins (30 µg) were separated using SDS-PAGE gels. Then the proteins were transferred to polyvinylidene difluoride (PVDF) membranes (Bio-Rad Laboratories). The PVDF membranes were then blocked with 5% BSA diluted in TBS for 1 h at room temperature. Primary antibodies against CCR1 (Invitrogen), total JNK (Affinity), p-JNK (Affinity), c-Jun (Affinity), p-p38 MAPK (Affinity), total p38 MAPK (Affinity) and β-actin (Affinity) were then added according to the manufacturers' protocols. The samples were agitated at 4 °C overnight. Goat anti-rabbit IgG HRP-conjugated secondary antibodies (ZSGB Bio, China) was added and incubated at room temperature for 2 h. The densitometric analysis was performed using ChemiDoc Touch Imaging System (Bio-Rad Laboratories).

### In vitro osteoclastogenesis assays

To induce osteoclast differentiation, CD115(+) early OCPs were exposed to induction medium consisting of MEM with 10% FBS, RANKL (100 ng/ml) and CSF (50 ng/ml) for at least 4 days with treatment of CCL7 (100 ng/mL). For tartrate-resistant acid phosphatase (TRAP) staining, induced cells were fixed in 4% paraformaldehyde for 10 min and then stained with TRAP staining solution according to the manufacturer’s instructions (Wako). Images were captured by an IX81 fluorescence microscope (Olympus).

### Flow cytometry analysis

To detect the percentage of early OCPs, bone marrow cells were flushed from the tibia at 10 days post injection of MC-38 cells and processed as described above, and then the cells were incubated in the appropriate primary antibodies for 30 min at 4 °C. The stained samples were analyzed by flow cytometry (BD FACSCalibur, BD Biosciences). The data were analyzed by using FlowJo v10 software (FlowJo, LLC).

### ELISA assays

Bone marrow from tibias at indicated timepoints were flushed out using PBS. The supernatant was collected after centrifugation. Equal volume of supernatant in each group was used for subsequent ELISA assay. The detection of protein level of CCL2, CCL6 and CCL7 was analyzed using ELISA detection kit (Novus, USA) following manufacturer’s protocol. The absorbency was detected at 450 nm.

### Histochemistry

Tibias from mice injected with MC-38 cells for 14 days, 21 days or 28 days were collected. Tibias were fixed in 4% paraformaldehyde (PFA) for 4 days, then the samples were washed and decalcified in a solution of 10% EDTA for 2 weeks and embedded in paraffin. Sections were stained with tartrate resistant acid phosphatase (TRAP) staining (Wako) or Safranin O and fast green staining following manufacturer’s procedures. Briefly, 0.5 mL of TRAP stain solution was added on each section and incubated for 30 min at RT. 0.1 M AMPD-HCl buffer (pH 9.4) was added to soak the sections for 10 min. For Safranin O-Fast Green staining, sections were immersed in 0.1% Safranin O solution for 3 min following by immersed in 0.1% Fast Green solution for 10 s. 1% acetum was utilized for color separation. The images were captured by a fluorescence microscope IX81 (Olympus, Japan).

### Proliferation assays

Phase-Flow BrdU kit (Biolegend) was used and the procedures were performed following manufacturer’s instruction. Briefly, BrdU solution was added into culture medium at a final concentration of 10 μg/mL and cultured for 2 h before collecting samples. The cells were collected, fixed, permeated and treated by DNase for 1 h at 37 °C. BrdU antibody (1:100) was added and treated for 30 min at 4 °C in dark.

### Apoptosis assays

In Situ Apoptosis Detection Kit (Roche) was used and the procedures were following the manufacturer’s instructions. Briefly, cell suspension was stained for extracellular markers of OCPs. Then, samples were incubated in TUNEL mixture for 1 h at 37 °C after fixed and permeated. After washing with PBS to remove excessive staining solution, the cell suspension was analyzed by flow cytometry as described above.

### Statistical analysis

Experiments were repeated at least 3 times. Results are presented as means ± SD. Student's *t*-test was used in comparison of two groups. For more than two groups, one way analysis of variance (ANOVA) was used. Statistical significance was considered at *P* < 0.05.

## Results

### Blockage of CCL7 prevented osteolysis in bone metastasis of MC-38

**Fig. 1:**
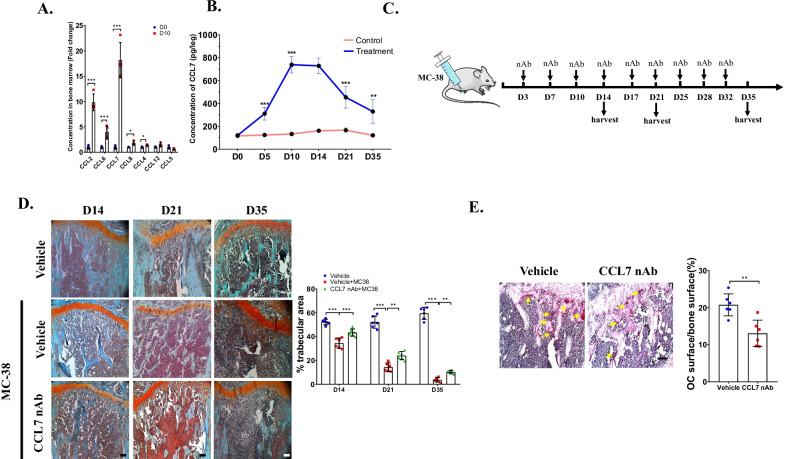
Blockage of CCL7 inhibiting osteolysis in bone metastasis of MC-38. **A** ELISA assay detected concentration of members of CCL family in bone marrow at 10 days post injection of MC-38. n = 4, each sample was pooled from 3 mice. **B** ELISA assay detected concentration of CCL7 in bone marrow at each timepoints after injection of MC-38 or vehicle. n = 8. **C** Schematics of treatment with CCL7 nAb after injecting of MC-38 cells. **D** Safranin O and fast green staining showed the trabecular area at 14 dpi, 21 dpi and 35 dpi after injection of MC-38 or not, with treatment with CCL7 nAb or vehicle (Scale bar = 50 μm) and quantification of trabecular area. n = 6. **E** TRAP staining showed OC surface at 14 days post injection of MC-38 after treatment with CCL7 nAb or vehicle (Scale bar = 50 μm) and quantification of OC surface/trabecular area. n = 6. **p < 0.01, ***p < 0.001

First, we investigated how the expression of several important members of CCL family was impacted during bone metastasis of MC-38. ELISA assay demonstrated that the protein levels of CCL2, CCL6, CCL7, CCL4 and CCL8 all upregulated significantly at 10 days post injection (dpi) of MC-38 compared to those in normal mice. Notably, the fold change of CCL7 increased highest among these three members, indicating CCL7 was the most significantly impacted member during bone metastasis of MC-38 cells (Fig. 1A). Then, we detected the trajectory of the expression of CCL7 during bone metastasis of MC-38 cells, the results showed the protein level of CCL7 upregulated once MC-38 cells were injected intratibially and reached the peak at 10 dpi (Fig. 1B), implying that CCL7 involved the bone metastasis of MC-38 at early stage. We next investigated whether blockage of endogenous CCL7 could impact osteolysis after bone metastasis (Fig. 1C). Histochemistry analysis revealed that the trabecular area was significantly restored at 14 dpi, 21 dpi and 35 dpi of MC-38 cells after treatment with CCL7 neutralizing antibody (nAb) (Fig. 1D). In addition, TRAP staining demonstrated that the area of osteoclasts (OCs) significantly decreased in CCL7 nAb treated group at 14 dpi. These results indicated that CCL7 participated in bone metastasis of MC-38 cells and involved in bone resorption through regulating the number of OCs.

### CCL7 promoted migration of OCPs

**Fig. 2:**
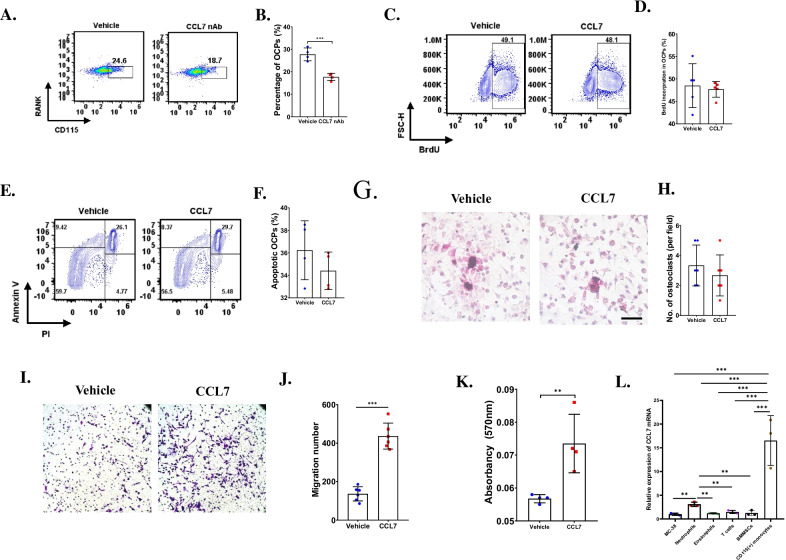
CCL7 promoted recruitment of early OCPs. **A**, **B** Flow cytometry analysis revealed percentage of CD115(+) early OCPs in bone marrow at 10 days post injection of MC-38 with treatment of CCL7 nAb or vehicle (**A**) and quantification of percentage of early OCPs (B). n = 4, each sample was pooled from 5 mice. **C**, **D** Flow cytometry analysis showed the BrdU incorporation in early OCPs treated by recombinant CCL7 or vehicle in the presence of MC-38 CM (**C**) and quantification of BrdU incorporation in early OCPs (**D**). n = 5. **E**, **F** Annexin V/PI analysis showed the apoptotic early OCPs treated by recombinant CCL7 or vehicle in the presence of MC-38 CM (**E**) and quantification of apoptosis of early OCPs (**F**). n = 4. **G**, **H** TRAP staining showed OCs after treated with CCL7 or vehicle in the presence of M-CSF (50 ng/mL) and RANKL (100 ng/mL) for 4 days (**G**) and quantification of OCs per field (H). n = 6. **I**, **J** Transwell analysis showed migration of early OCPs treated by CCL7 for 12 h (**I**) and quantification of migration number of early OCPs (**J**). n = 6. **K** Quantification of **I** using Absorbency analysis. n = 4. **L** qRT-PCR analysis detected mRNA level of CCL7 in neutrophils, eosinophils, T cells, BMMSCs and early OCPs isolated from 10 days post injection of MC-38 compared to that in MC-38 cells. n = 3, each sample was pooled from 5 to 8 mice. **p < 0.01, ***p < 0.001

Next, we explored how CCL7 regulated the number of OCs. After injection of CCL7 nAb, we detected the percentage of CD115 ( +) monocytes (early OCPs) at 10 dpi. Interestingly, the percentage of early OCPs significantly decreased in CCL7 nAb treated group (Fig. 2A, B), indicating that CCL7 could impact the number of early OCPs. Then, we investigated how early OCPs were regulated by CCL7. Early OCPs were isolated through FACS and cultured with MC-38 CM with treatment of CCL7 or not. The BrdU incorporation of early OCPs showed no significant difference between control group and CCL7 treated group (Fig. 2C, D). In addition, Annexin V/PI assay revealed that the percentage of apoptotic early OCPs was not significantly changed after treatment with CCL7 (Fig. 2E, F). These results together indicated that CCL7 did not regulate the proliferation and apoptosis of early OCPs in CRC microenvironment. We next explored whether osteoclastogenesis could be directly regulated by CCL7, TRAP staining revealed that the number of OCs showed no significant difference between control group and CCL7 treated group (Fig. 2G, H), demonstrating that CCL7 also did not regulate osteoclast formation. Because CCL7 was reported to be a chemokine which recruited monocytes. Then, we further investigated whether the migration of early OCPs could be regulated by CCL7. Interestingly, Transwell assay showed the CCL7 significantly recruited early OCPs (Fig. 2I–K). These data indicated that the number of early OCPs could be regulated by CCL7 through promoting the migration.

### Early OCPs were the dominant source of CCL7

To explore the potential cellular source of CCL7 in bone marrow after bone metastasis of MC-38, several important cell types, including early OCPs, bone marrow derived mesenchymal stem cells (BMMSCs), neutrophils, eosinophils, T cells were isolated through FACs from bone marrow in normal mice and in mice at 10 dpi (Additional file 1: Fig S1A–S1D). Then, the mRNA level of CCL7 in these cell types as well as MC-38 cells was compared with each other. The results showed that the mRNA level of CCL7 significantly upregulated in early OCPs isolated from bone marrow at 10 dpi, and the fold change of early OCPs was highest among these cell types (Fig. 2L). These results indicated that early OCPs were the dominant cellular source of CCL7 in CRC microenvironment.

### Blockage of CCL7 prevented the recruitment of early OCPs

**Fig. 3:**
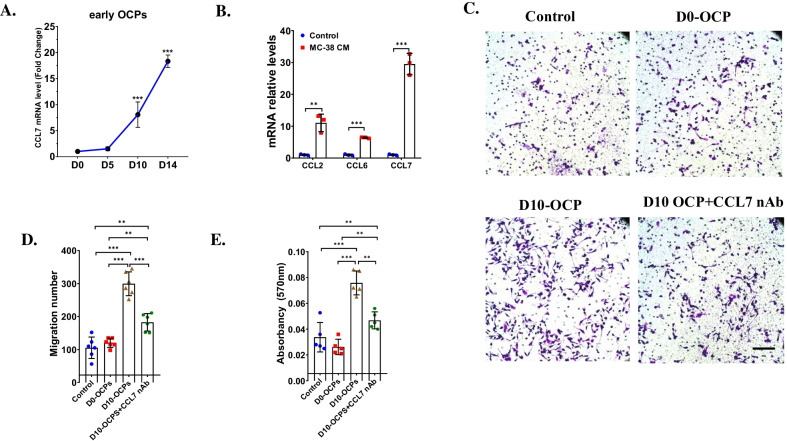
Blockage of CCL7 inhibited migration of early OCPs. **A** qRT-PCR analysis showed mRNA level of CCL7 in primary early OCPs isolated from bone marrow at each timepoints after injection of MC-38. n = 3. **B** qRT-PCR analysis detected mRNA levels of CCL2, CCL6 and CCL7 in early OCPs cultured in MC-38 CM. n = 3. **C**, **D** Transwell analysis showed migration of early OCPs indirectly cultured with early OCPs isolated from bone marrow in normal mice or in mice at 10 days post injection of MC-38 with/without treatment of CCL7 nAb (**C**) and quantification of migration number of early OCPs (**D**). n = 6. **E** Quantification of **C** using Absorbency analysis. n = 5. **p < 0.01, ***p < 0.001

We next further investigated the expression of CCL7 in early OCPs during bone metastasis of MC-38. The mRNA level of CCL7 in early OCPs upregulated from the beginning of injection of MC-38 (Fig. 3A), which was similar with the trajectory of the protein level of CCL7 in bone marrow in bone metastasis of MC-38. Then, we compared whether other important CCL family members produced by early OCPs could also be impacted by CRC microenvironment, qRT-PCR analysis demonstrated that the expression of CCL7 upregulated most significantly in early OCPs after treated by MC-38 CM compared to that of CCL2 and CCL6, indicating early OCPs dominantly produced CCL7 after stimulated by MC-38 secreta (Fig. 3B).

Then, we verified whether early OCPs in tumor microenvironment could recruit more early OCPs through a CCL7-dependent way. As expected, no significant difference of early OCP migration can be found between the control group and D0-early OCPs group, however, secreta from D10-early OCPs can recruit much more early OCPs than that of control group and D0-early OCPs group. However, when treating with CCL7 nAb, the effect of D10-early OCPs on recruitment of early OCPs was significantly prevented (Fig. 3C–E). These results indicated that CCL7 was a critical regulator in tumor microenvironment to promote migration of early OCPs.

### CCL7 was upregulated by Lactate through JNK pathway

**Fig. 4:**
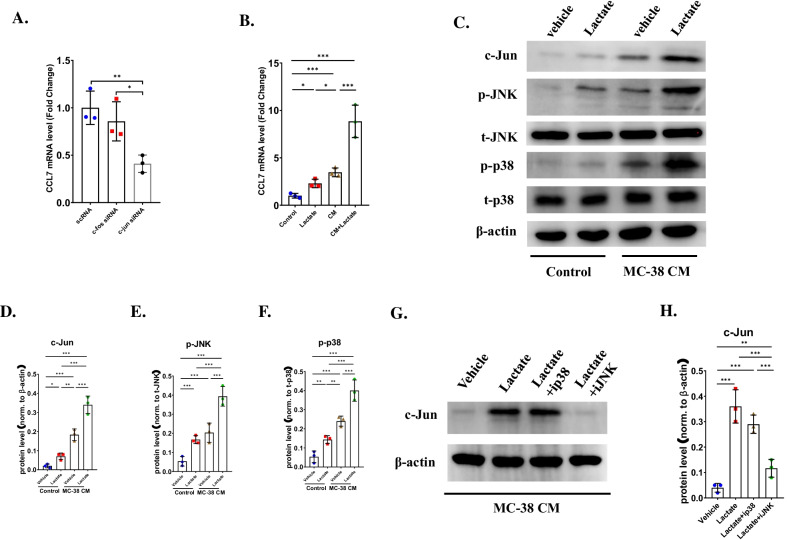
Lactate upregulated expression of c-Jun through activating JNK pathway. **A** qRT-PCR analysis detected mRNA level of CCL7 in early OCPs after transfection with c-Fos siRNA or c-Jun siRNA. n = 3. **B** qRT-PCR analysis detected mRNA level of CCL7 in early OCPs after treatment of lactate in the presence of MC-38 CM or not. n = 3. **C**–**F** Western blotting analysis showed protein levels of c-Jun, JNK, p38 MAPK and their total protein in early OCPs after stimulated with lactate in the presence of MC-38 CM or not (**B**) and the quantification of protein level normalized to β-actin (**C**–**E**). n = 3. **G**, **H** Western blotting analysis showed protein levels of c-Jun in early OCPs after stimulated with lactate plus p38 MAPK antagonist or JNK antagonist in the presence of MC-38 CM (**F**) and the quantification of protein level normalized to β-actin (**G**). n = 3. *p < 0.05, **p < 0.01, ***p < 0.001

AP-1 was reported to be a transcription factor of CCL7, thus we investigated whether c-Jun or c-Fos regulated the expression of CCL7 in OCPs. qRT-PCR analysis showed that the mRNA level of CCL7 can be significantly downregulated after transfection with c-Jun siRNA, but not c-Fos siRNA (Fig. 4A). These results indicated that c-Jun may be a transcription factor of CCL7 in OCPs in CRC microenvironment. In our previous study, we demonstrated that MC-38 could produce abundant lactate. Interestingly, lactate was reported to active MAPK pathways. Thus, we explored whether the expression of c-Jun could be regulated by lactate through MAPK pathway. Firstly, we detected whether lactate stimulated the expression of CCL7. As expected, qRT-PCR analysis showed that administration of lactate could upregulate the mRNA level of CCL7. Notably, more significant upregulation could be observed in the presence of MC-38 CM (Fig. 4B). These results indicated that lactate could regulate the expression of CCL7 in early OCPs. Then, we detected whether lactate regulated the expression of c-Jun. Western blotting revealed that the expression of c-Jun upregulated after treatment with MC-38 CM (Fig. 4C, D). In addition, both JNK pathway and p38 MAPK pathway was activated after treatment of lactate, especially in the presence of MC-38 CM (Fig. 4C–F). In addition, this trajectory could be further enhanced while OPCs were cultured in MC-38 CM (Fig. 4C, D). These results showed that lactate could induce the expression of c-Jun. Then, we further investigated whether p38 MAPK or JNK pathway regulated the expression of c-Jun in OCPs. Early OCPs cultured in MC-38 CM were treated by lactate with/without p38 MAPK antagonist or JNK antagonist. Western blotting indicated that only the protein level decreased in OCPs treated by JNK antagonist, but not p38 MAPK antagonist (Fig. 4G, H), indicated that JNK pathway was the upstream of c-Jun in OCPs after treatment of lactate in CRC microenvironment.

### CCR1 involved in CCL-7 medicated migration of OCPs

**Fig. 5:**
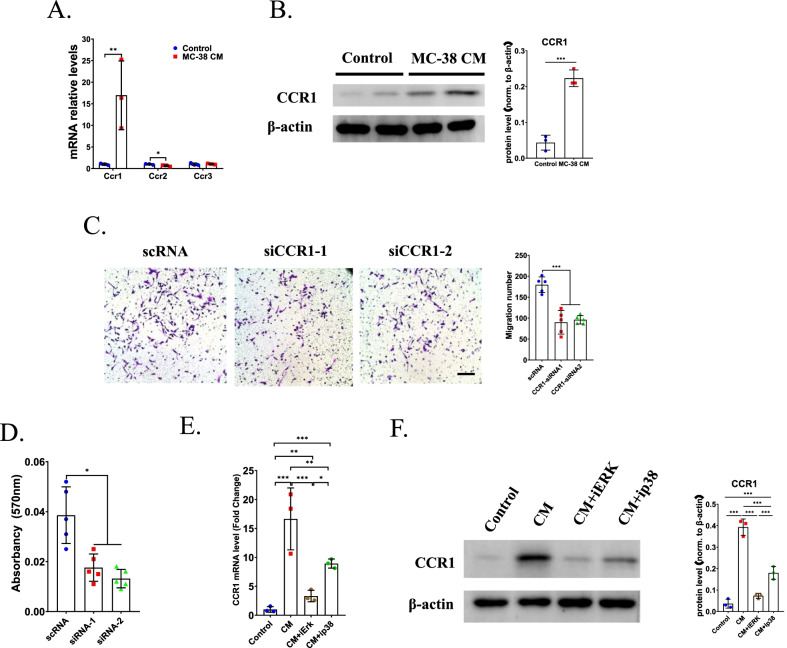
CCR1 upregulated in early OCPs in CRC microenvironment. **A** qRT-PCR analysis detected mRNA level of CCR1, CCR2 and CCR3 in early OCPs cultured in MC-38 CM. n = 3. **B** Western blotting analysis showed protein levels of CCR1 in early OCPs cultured in MC-38 CM or not (left) and the quantification of protein level normalized to β-actin (right). n = 3. **C** Transwell analysis showed migration of early OCPs transfected with CCR1 siRNA or scRNA in the presence of CCL7 (left) and quantification of migration number of early OCPs (right). n = 5. **D** Quantification of **C** using Absorbency analysis. n = 5. **E** qRT-PCR analysis detected mRNA level of CCR1 in early OCPs cultured in MC-38 CM with/without treatment of ERK antagonist or p38 MAPK antagonist. n = 3. **F** Western blotting analysis showed protein levels of CCR1 in early OCPs cultured in MC-38 with/without treatment of p38 MAPK antagonist or ERK antagonist (left) and the quantification of protein level normalized to β-actin (right). *p < 0.05, **p < 0.01, ***p < 0.001

CCR1, CCR2 and CCR3 were reported to be the receptors of CCL7, thus we next investigated which one of them regulated the CCL7-mediated migration of OCPs. qRT-PCR showed that the mRNA level of CCR1 significantly upregulated in OCPs cultured in MC-38 CM compared to that of CCR2 and CCR3 (Fig. 5A). In addition, the protein level of CCR1 in OCPs upregulated gradually with the increased radio of MC-38 CM and DMEM (Fig. 5B), implying that CCR1 may be the dominant receptor of CCL7 in OCPs in CRC microenvironment. Consistently, the migration of OCPs significantly prevented after transfection with CCR1 siRNA (Fig. 5C, D). Next, we explored how the expression of CCR1 was regulated in CRC microenvironment. qRT-PCR showed that treatment with MC-38 CM significantly upregulated the mRNA level of CCR1, however, both inhibiting the activation of ERK pathway and p38 MAPK pathway could reverse this effect, demonstrating that both two pathways regulated the expression of CCR1 in OCPs (Fig. 5E). Consistently, the protein level of CCR1 could be induced by MC-38 CM, however, treatment with ERK antagonist or p38 MAPK antagonist could significantly decrease the protein level of CCR1 (Fig. 5F).

### Targeting CCR1 attenuated bone resorption in bone metastasis of MC-38

**Fig. 6:**
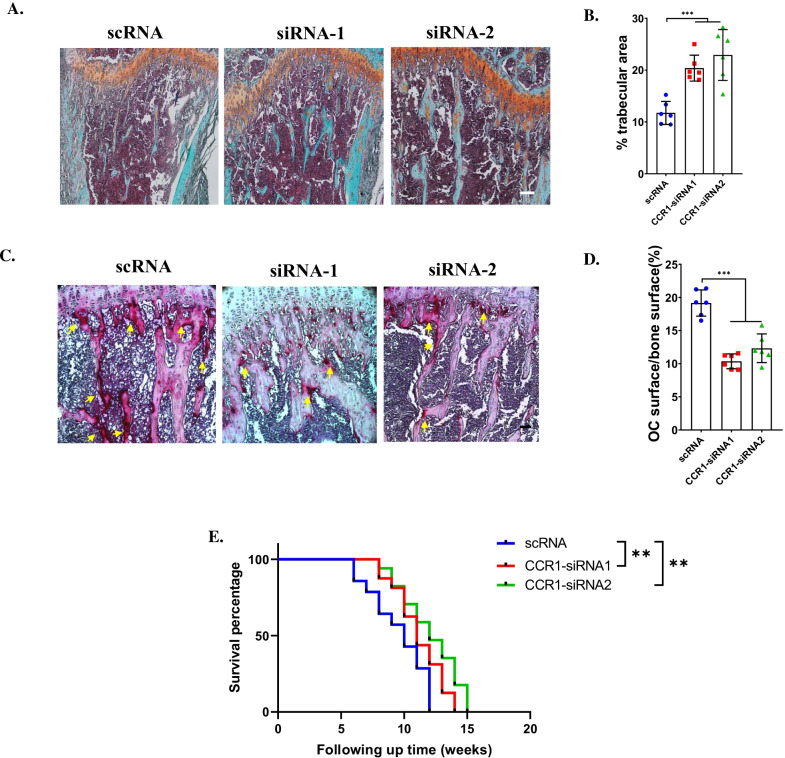
Treatment of CCR1 siRNA attenuated osteolysis in bone metastasis of MC-38. **A**, **B** Safranin O and fast green staining showed the trabecular area at 14 days post injection of MC-38 after treatment with CCR1 siRNA or scRNA (Scale bar = 50 μm) (**A**) and quantification of trabecular area (**B**). n = 6. **C**, **D** TRAP staining showed OC surface at 14 days post injection of MC-38 after treatment with CCR1 siRNA or scRNA (Scale bar = 50 μm) (**C**) and quantification of OC surface/trabecular area (**D**). n = 6. **E** Prognosis of mice after treatment with siCCR1 after injection of MC-38 cells. ***p < 0.001

To investigate whether CCR1 could be a potential therapeutical target for treating bone metastasis of CRC, CCR1 siRNA were injected intratibially. Histochemistry analysis showed that trabecular area was restored after treatment with CCR1 siRNA at 14dpi (Fig. 6A, B). In addition, the osteoclast area was significantly decreased in CCR1 siRNA-treated group (Fig. 6C, D). To evaluate the prognosis, survival curve showed that treatment with siCCR1 could significantly prolong the lifetime (Fig. 6E).

## Discussion

In this study, we explored that CCL7 was a key regulator for recruitment of early OCPs. Furthermore, we unexpectedly found early OCPs in tumor microenvironment were the dominant source for CCL7. A positive feedback loop for recruiting abundant early OCPs to metastatic sites was formed through secreting CCL7.

Since osteoclasts derived from monocyte/macrophage lineage, the number of monocytes/macrophages determined the number of OCs. It is reasonable that monocyte chemoattractant proteins may play a key role in bone metastasis of osteolytic tumors. Indeed, several studies emphasized the potential role of CXC chemokine ligands in promoting the tumor metastasis. CCL2, a dominant monocyte chemoattractant protein to recruit macrophages through binding to CCR2, was found to be a key mediator for osteoclastogenesis in bone metastasis of breast cancer and prostate cancer [18]. Eosinophils were reported to be responsible for promoting bone metastasis through producing CCL6 [19]. In addition, increased expression of CCL7 was associated with higher incidence of bone metastasis of non-small cell lung cancer cells [12]. In this study, we found CCL2, CCL6 and CCL7 all upregulated in bone marrow after injection of MC-38 cells, indicating that multiple monocyte chemoattractant proteins involved in bone metastasis of CRC. Among them, the upregulation of CCL7 was highest, implying a dominant role during bone metastasis of MC-38 cells. However, we did not find that CCL7 could directly regulate osteoclastogenic differentiation, promote proliferation or inhibit apoptosis of OCPs in vitro, thus CCL7 may involve in the increased number of OCs dominantly dependent on recruitment of OCPs. Similarly, CCL7 was reported to enhance the migration of RAW264.7, a commonly used monocyte/macrophage cell line, which supported our findings [20]. Interestingly, many studies indicated that CCL7 could promote metastasis of CRC, such as lung metastasis, through JAK-STAT pathway and ERK-JNK pathway [21–23]. Consistently, we here explored that CCL7 played a key role in the bone metastasis of CRC. Our findings further revealed the importance of CCL7 in metastasis of CRC.

We here found lactate could promote the expression of CCL7 in early OCPs through JNK pathway. It was reported that high active Warburg effect could be detected in colorectal cancer cells [24]. In our previous study, we found lactate could promote the production of CXCL10 in early OCPs to recruit CD4+ T cells [25]. Together with this study, these results indicated that lactate may broadly modulate the secreting spectrum of chemoattractant proteins to facilitate the formation of metastatic microenvironment. In addition, AP1 was predicted to be the transcription factor of CCL7, however, we here only found c-Jun could significantly regulate the mRNA level of CCL7. MAPK pathway could be activated by lactate [26], we here found both p38 MAPK and JNK pathway can be activated by lactate, especially in the presence of MC-38 CM. Blockage of these two pathways can both downregulate the expression of CCR1, indicating that lactate regulated the expression of CCR1 through at least these two pathways.

CCR1, CCR2 and CCR3 were reported to be potential receptors of CCL7 in tumor microenvironment [27]. In this study, we found that the expression of CCR1 upregulated remarkedly compared with the expression of CCR2 or CCR3, indicating that CCR1 may be the dominant receptor of early OCPs in CRC microenvironment. Downregulation of CCR1 could significantly prevent CCL7-mediated migration of early OCPs in bone metastasis of CRC. Our findings demonstrated that CCL7-CCR1 could be the most important signal to regulate migration of early OCPs at least in CRC microenvironment. Similarly, it was also reported that CCR1 involved CCL7-induced liver metastasis of CRC as well as gastric cancer metastasis [13, 28]. These findings together indicated that CCL7-CCR1 could be an important way for tumor metastasis. In addition, we also found the expression of CCR1 could be significantly upregulated by MC-38 CM, however, it is still unclear which component(s) in MC-38 derived secreta regulated the upregulation of CCR1 in OCPs, which should be further investigated.

## Conclusion

In this study, we explored that CCL7 was an important regulator for recruitment of early OCPs in an autocrine manner during bone metastasis of CRC. Mechanically. CCL7 promoted the migration of early OCPs dominantly through CCR1. Lactate could stimulate the upregulation of CCL7 in early OCPs through activation of JNK pathway. Targeting CCL7 or CCR1 could significantly prevent bone resorption caused by CRC cells.

## Supplementary Information

**Additional file 1. Figure S1: **Early OCPs were the primary cellular source of CCL7 in CRC-microenvironment. (A) Schematic of sorting dominant cellular types from bone marrow at 10 days post injection of MC-38 cells. (B) Representative image of flow cytometry strategy of isolating neutrophils and eosinophils. (C) Representative image of flow cytometry strategy of isolating T cells. (D) Representative image of flow cytometry strategy of isolating BMMSCs. (E) qRT-PCR analysis detected mRNA level of CCL7 in neutrophils, eosinophils, T cells, BMMSCs and early OCPs isolated from 10 days post injection of MC-38 compared to that in MC-38 cells. n=3, each sample was pooled from 5-8 mice. **p<0.01, ***p<0.001.

## Data Availability

All data generated or analyzed during this study are included in this published article and its supplementary information files.

## Abbreviations

CCL: Chemokine (C–C motif) ligand
CCR: Chemokine (C–C motif) receptor
CRC: Colorectal cancer
OCP: Osteoclast precursor
CM: Conditional medium
CXCL: Chemokine (C–X–C motif) ligand
MAPK: Mitogen-activated protein kinase
JNK: C-Jun N-terminal kinase
PFA: Paraformaldehyde
nAb: Neutralizing antibody
TRAP: Tartrate resistant acid phosphatase
DMEM: Dulbecco's modified Eagle's medium
BMMSCs: Bone marrow derived mesenchymal stem cells
