# Supplementary material for: CCL7 playing a dominant role in recruiting early OCPs to facilitate osteolysis at metastatic site of colorectal cancer
Source: Cell Commun Signal. 2022 Jun 17;20:94. doi: 10.1186/s12964-022-00867-7 (PMC9205124; doi:10.1186/s12964-022-00867-7)
Supplement: Supplementary file 2 — Additional file 1. Figure S1: Early OCPs were the primary cellular source of CCL7 in CRC-microenvironment. (A) Schematic of sorting dominant cellular types from bone marrow at 10 days post injection of MC-38 cells. (B) Representative image of flow cytometry strategy of isolating neutrophils and eosinophils. (C) Representative image of flow cytometry strategy of isolating T cells. (D) Representative image of flow cytometry strategy of isolating BMMSCs. (E) qRT-PCR analysis detected mRNA level of CCL7 in neutrophils, eosinophils, T cells, BMMSCs and early OCPs isolated from 10 days post injection of MC-38 compared to that in MC-38 cells. n=3, each sample was pooled from 5-8 mice. **p<0.01, ***p<0.001. [file 12964_2022_867_MOESM2_ESM.pptx]

## Slide 1
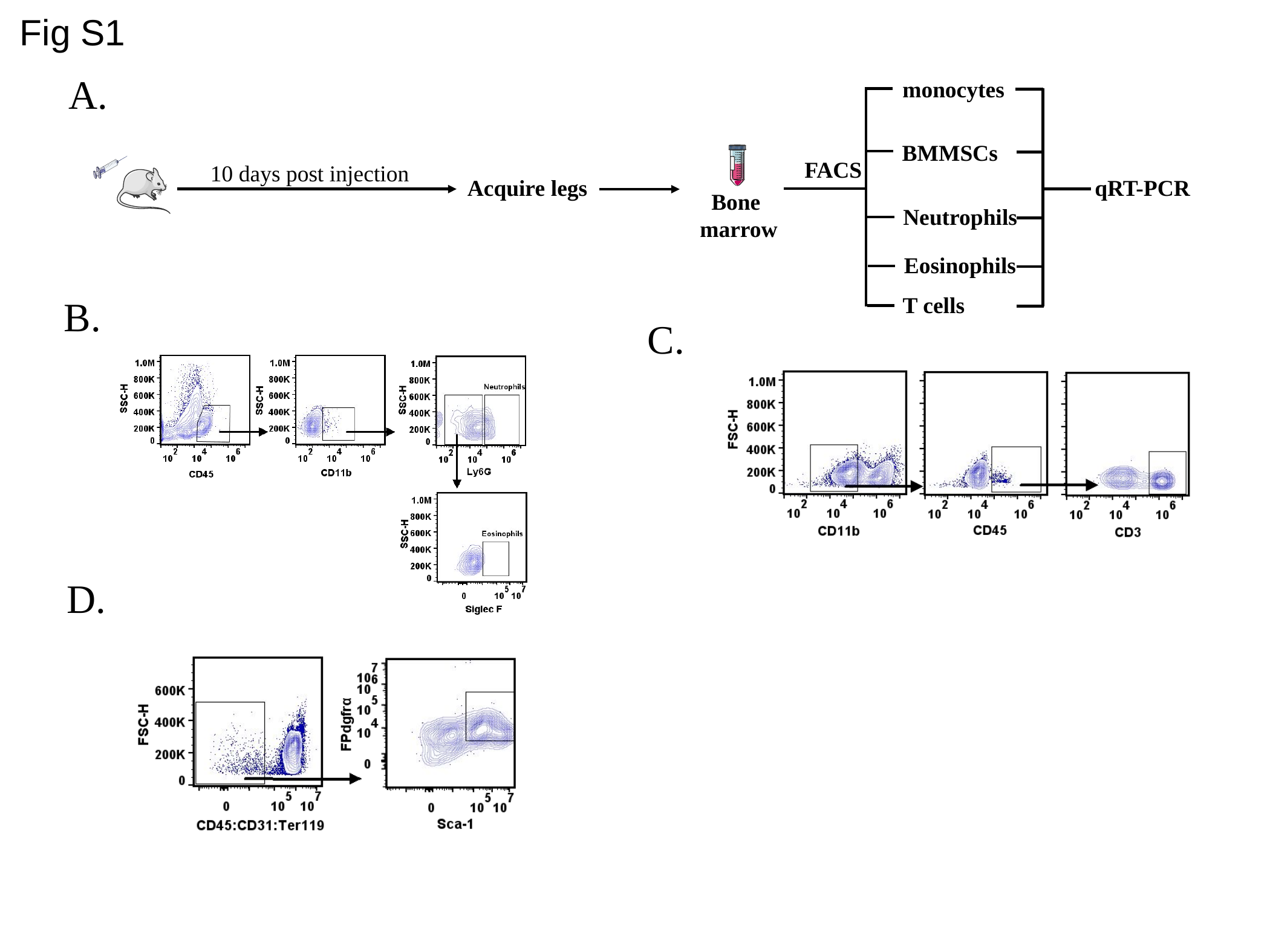

Fig S1
A.
monocytes
BMMSCs
FACS
10 days post injection
Acquire legs
qRT-PCR
Bone
 marrow
Neutrophils
Eosinophils
T cells
B.
C.
D.
